# Report on the first detection of Asian citrus psyllid *Diaphorina citri* Kuwayama (Hemiptera: Liviidae) in the Republic of Benin, West Africa

**DOI:** 10.1038/s41598-023-28030-3

**Published:** 2023-01-16

**Authors:** Mamoudou Sétamou, Yovanna L. Soto, Martine Tachin, Olufemi J. Alabi

**Affiliations:** 1 Texas A&M University-Kingsville Citrus Center, Weslaco, 78599 USA; 2 School of Horticulture and Management of Green Spaces, National University of Agriculture (UNA), Kétou, Republic of Benin; 3 Department of Plant Pathology & Microbiology, Texas A&M AgriLife Research & Extension Center, Weslaco, TX 78596 USA

**Keywords:** Genomic analysis, Plant sciences, Entomology

## Abstract

The Asian citrus psyllid (ACP), *Diaphorina citri*, was detected for the first time in the Republic of Benin, West Africa. The ACP is a known vector of *Candidatus* Liberibacter asiaticus (CLas), the putative causal agent of the devastating Huanglongbing (HLB; citrus greening disease). During visual surveys, ACP was only observed on residential citrus trees in southern Benin, but not in residential areas or commercial groves in the central and northern parts of the country. Its identity was confirmed morphologically and molecularly via DNA barcoding with published primers. Analysis of the obtained sequences showed that the ACP recorded in Benin clustered with the ones previously reported from Nigeria, suggesting a common origin of both populations. The ACP samples from Benin also carried *Ca*. Carsonella ruddii and *Ca*. Profftella armatura, two commonly found ACP endosymbionts. However, all the sampled ACP individuals tested negative for *Ca*. Liberibacter africanus, *Ca*. Liberibacter americanus, and CLas by quantitative polymerase chain reaction. This is the second report of the ACP in West Africa after Nigeria, the eastern bordering country of the Republic of Benin. Benin has an expanding commercial citrus industry, especially in the southern part of the country. Although the ACP samples tested negative for the HLB associated bacteria, the detection of ACP in the country requires swift actions including area-wide surveys to determine the extent of spread of this pest and the implementation of eradication or control efforts to prevent its establishment and spread of HLB in the country.

## Introduction

In the Republic of Benin, citrus is grown commercially as a cash crop and as backyard tree like in many other parts of the world. Commercial citrus production in the country started soon after the independence in the 1960s, with the creation of the national fruits and vegetables company (Société Nationale des Fruits and Légumes, SoNaFeL) via a Benin-Israeli cooperation^1^. From 1960 to the 1970s, several citrus varieties of lime, lemon, grapefruit, mandarin, sweet oranges and tangelo were introduced for the establishment of commercial groves mainly in the southern part of the country^2–5^, but sweet orange remained the dominant commercial citrus species in the Republic of Benin to date^6^.

From the onset of commercial citrus industry in Benin, the planted acreage slowly increased and reached ca. 2500 ha of sweet oranges in 1977 where it plateaued until 1989. Commercial citrus acreage declined from 1989 to 1992, as a consequence of the closure in 1986 of SoNaFeL because the country underwent the World Bank and International Monetary Fund structural adjustment programs^7^. Subsequently, the harvested commercial orange acreage has rebounded and gradually increased reaching ca. 6500 ha in 2020^6^. Despite the apparent expansion in acreage in recent years mainly due to the implantation of a processing plant in the commercial citrus belt of Benin, citrus yields in Benin remain among the lowest in the world. In 2020, average orange yields were estimated at 2.5 MT ha^−1^, the second lowest commercial yields recorded in Africa and far below the world average of 14–15 MT ha^−1^^6^. Several biotic and abiotic factors are responsible for these meager citrus yields in Benin. Firstly, commercial citrus groves in Benin have no irrigation systems and trees are only rain fed. Erratic rainfall can negatively affect citrus yields and production^8,9^, as citrus trees require adequate water supply for fruit sizing. In addition, citrus groves in Benin remain largely unmanaged due to poor access to inputs including fertilizers and agrochemicals^10^. Under these conditions, pests and diseases can develop unchecked leading to substantial damage and poor yields. Indeed, over 80% of commercial citrus growers in Benin list the lack of agricultural inputs and pressure from pests and diseases as major constraints to citrus production, with arthropod pests and diseases reported as the most important impediments^10^. The hot and humid tropical climate prevailing in the citrus production areas of Benin favor the establishment and spread of many arthropod pests and diseases. Among the arthropod pests limiting citrus productivity in Benin, aphids (*Aphis gossypii* Glover and *Myzus persicae* (Sulzer)), the citrus leafminer (*Phyllocnistis citrella* Stainton), the grasshopper *Zonocerus variegatus* (Linnaeus), the green tree ant *Oecophylla smaragdina* (Fabricius), the cottony cushion scale *Icerya purchasi* Maskel, the citrus rust mite *Phyllocoptruta oleivora* (Ashmead) and fruit piercing moths (*Othreis fullonia* (Clerk), *Ophideres sp*., *Achaeasp sp*. and *Enmonodia sp*.) are cited as of great concern^2,3,7,11–13^. Zadji^14^ reported a complex of termites (*Trinervitermes occidentalis* (Sjöstedt), *Amitermes guineensis* (Sjöstedt), *Macrotermes bellicosus* (Smeathman), and *Ancistrotermes crucifer* (Sjöstedt) as the most devastating group of arthropod pests, boring galleries into stems, thus weakening trees and reducing their productivity by 25–50%. Vayssieres et al.^12^ listed dipteran fruit flies (*Bactrocera. invadens* Drew, Tsuruta & White, *B. cucurbitae*, *Ceratitis fasciventris* (Bezzi), *C. ditissima* (Munro), *C. anonae* Graham, and *Dacus punctatifrons* Karsch), as pests of significant importance causing substantial losses of fruit quality and about 35% loss of total production in sweet orange varieties.

In addition to direct feeding damage, some arthropod pests are recognized vectors of pathogens causing economically damaging citrus diseases. One such pathogen, the citrus tristeza virus (CTV), is the most destructive virus known to citrus production^15^, and its efficient vector the brown citrus aphid (*Toxoptera aurantii* (Boyer de Fonscolombe)) is endemic in Benin^5,13^. Despite the economic importance of CTV in citriculture, Huanglongbing (HLB) or citrus greening is currently the most devastating citrus disease worldwide^16^. HLB is a destructive disease that has spread into major citrus production areas in the Americas and Africa^16–19^. HLB is putatively caused by three related phloem-inhabiting fastidious bacteria namely *Candidatus* Liberibacter africanus (CLaf), *Ca*. Liberibacter americanus (CLam) and *Ca*. Liberibacter asiaticus (CLas)^16^. These bacteria are spread via vegetative propagation of infected plant materials and by the two psyllid vectors, the African citrus triozid (ACT, *Trioza erytreae* (Del Guercio) (Hemiptera: Triozidae)) and the Asian citrus psyllid (ACP, *Diaphorina citri* Kuwayama (Hemiptera: Liviidae)). While ACT is known to specifically transmit CLaf restricted to the African continent, CLam and CLas are spread by ACP^20^. CLam is known to occur exclusively in the Americas, but CLas has spread across continents, currently occurring in major citrus producing countries in Africa, America and Asia^19,21–23^.

*Diaphorina citri* is an invasive and fast spreading pest. During the past few decades, ACP has invaded several countries and states in the Americas^22,24,25^ and Africa^21,26–28^. ACP presence in sub-Saharan Africa represents a significant threat to the sustainability of citrus production in these countries, which is already crippled by several other biotic and abiotic production constraints. Although the presence of CLas in Africa is currently limited to East Africa^21,26,27^, the detection of ACP in Nigeria in West Africa^28^ points to a continent-wide potential of spread via trade and natural dispersal of this highly prolific vector.

Like ACP, ACT is an invasive species that is the main vector of CLaf, but has been shown to experimentally transmit CLas. Although ACT mainly occurs in eastern and southern Africa and the Middle East in Asia^29^, ACT has invaded Portugal and Spain in Europe in recent years^30,31^. However, its presence in West Africa is not fully elucidated.

Improving citrus yields and production will require mitigating the impacts of the many biotic factors currently identified as the most important production constraints. Given the presence of ACP in neighboring Nigeria^28^, and the high propensity of both ACP and ACT to spread and colonize new areas, studies are needed to determine their occurrence in the citrus producing areas of Benin. An early detection of ACP and ACT in an area is critical for the enactment of effective mitigation efforts. The presence of both psyllids in Benin has not been previously evaluated. This study was conducted to determine whether ACP and ACT and the HLB pathogens CLas and CLaf occur in residential and commercial citrus locales in Benin.

## Materials and methods

### Sample collection

A citrus pest survey was conducted at six residential sites and three commercial groves in December 2021 in five of the 12 departments of the Republic of Benin to assess the presence of *D. citri* and *T. erytreae* (Table 1). The residential sites were visited upon homeowners’ approval to evaluate the health of trees because of either poor growth or the presence of sooty mold. The commercial groves located in close proximity of the main highway were surveyed and sampled during a road trip. At each site, the host plant species was determined by evaluated the leaves and/or fruit when present, and the tree or grove location coordinates were recorded using the Compass app of an IPhone 12 pro^32^. All residential host plants present at the survey sites were visually examined for the presence of arthropod pests with special reference to the life stages of ACP or their wax-like feeding droppings on leaves^33^ and of ACT and the small pit galls it causes on young leaves^34^. In commercial groves, a sample of ten trees was randomly selected for evaluation. As young flush shoots are the breeding sites for ACP and ACT^34,35^, these flush shoots when present, were preferentially examined. Suspect ACP nymphs and adults were collected and stored in plastic vials containing 95% ethanol until further analysis and processing. No ACT was collected during the surveys, hence subsequent analyses focused on ACP.

**Table 1:** Survey location and suspect Asian citrus psyllid (*Diaphorina citri* Kuwayama) individuals collected from host trees Benin Republic, West Africa.

Location^a^	Host plants	Coordinate	Altitude (m)	Citrus system	Adults	Nymphs
Agblangandan, Oueme	Lime	N06°22ʹ23.95″ E02°29ʹ20.46″	6	Dooryard	3	0
Abomey Calavi, Atlantique	Sweet orange	N06°26ʹ04.73″ E02°20ʹ33.75″	18	Dooryard	12	10
Tankpe, Atlantique	Orange jasmine	N06°25ʹ54.12″ E02°19ʹ41.43″	18	Dooryard	4	0
Parakou, Borgou	Sweet orange, grapefruit, orange jasmine	N09°19ʹ07.17″ E02°35ʹ49.57″	391	Dooryard	0	0
Kalale, Borgou	Lime, tangerine	N10°17ʹ25.75″ E03°23ʹ00.92″	412	Dooryard	0	0
Kalale, Borgou	Sweet orange, lime	N10°17ʹ10.36″ E03°22ʹ49.84″	393	Dooryard	0	0
Setto, Zou	Valencia Sweet orange	N07°22ʹ52.74″ E02°04ʹ35.19″	170	Grove	0	0
Setto, Zou	Valencia Sweet orange	N07°23ʹ29.32″ E02°04ʹ30.74″	165	Grove	0	0
Cove, Zou	Valencia Sweet orange	N07°12ʹ18.17″ E02°11ʹ24.27″	109	Grove	0	0

^a^City, Department.

### Identification of *D. citri*

#### Morphological identification

All insect samples were brought to the Texas A&M University-Kingsville Citrus Center (TAMUK-CC) entomology laboratory for further analysis. Using the morphological characteristics described by Mead^33^ and Yang^36^, the identification of suspect nymphs and adults was made by comparing them to archived voucher specimens kept in the laboratory. Voucher specimens of identified *D. citri* from Benin are kept at TAMUK-CC.

#### Nucleic acid isolation and PCR

Total nucleic acids were extracted from morphologically identified *D. citri* adults and nymphs following the Dellaporta et al.^37^ protocol. A NanoDrop 2000 series spectrophotometer (Thermo Fisher Scientific Inc., Waltham, MA, USA) was used to quantify and analyze the quality of the extracted nucleic acids. After quantification and quality analysis, the total nucleic acid samples were stored at − 20 °C until when they were subjected to polymerase chain reaction (PCR) assays as described by Oke et al.^28^. Briefly, a 2 µL aliquot of each total nucleic acid sample was used as template in a 25 μL PCR with reagents and Rapid Protocol described for the PrimeSTAR GXLDNA Polymerase (Takara Bio USA, Inc., Mountain View, CA). PCR was performed on each insect total nucleic acid sample using three distinct primer pairs that target specific genes encoded by the ACP and two of its endosymbionts. The 834 bp fragment of the mtCOI coding region was targeted by the DCITRI COI-L (5′-AGGAGGTGGAGACCCAATCT-3′) and DCITRI COI-R (5′-TCAATTGGGGGAGAGTTTTG-3′) primer pair^38^. Additionally, single copy housekeeping gene-specific primer pairs argH-F1 (5′-CTCCTATGCCTGGATTTACTCA-3′) & argH-R1 (5′-TTGATTAGGCGCTGTACCTCC-3′) and atpA-F1 (5′-CAATAATCGGTATCGCTGTT-3′) & atpA-R1 (5′-AGCATATTACGGAAGGTGAT-3′) were used to target the *argH* of the psyllid primary endosymbiont (P-endosymbiont) *Ca* Carsonella ruddii and the *atpA* of the secondary endosymbiont (S-endosymbiont) *Ca* Profftella armatura, respectively^20^. The DNA amplicons from these samples were ran on 1% agarose gels pre-stained with ethidium bromide along with the 100–2000 bp Wide-Range DNA Ladder (Takara Bio USA, Inc.) and visualized under a UV-transilluminator. For all analyses, DNA extracts from laboratory colony of *D. citri* maintained at TAMUK-CC were included as positive controls.

#### Cloning and sequencing

Cloning and sequencing of DNA samples were conducted as described by Oke et al.^28^. Succinctly, target specific DNA bands of the correct sizes of the *D. citri* samples were excised and gel-eluted using the Zymoclean™ Gel DNA Recovery Kit (Zymo Research, Irvine, CA). Using the CloneJET PCR Cloning Kit (Thermo Fisher Scientific), the recovered DNA samples were ligated individually into the pJET1.2/blunt vector as per the manufacturer’s recommendations. Chemically competent DH5α *Escherichia coli* cells were transformed from the ligation products, and two to three plasmids with PCR-verified correct size inserts per cloned DNA amplicon were isolated from recombinant *E. coli* cells using the GenElute Plasmid Miniprep Kit (Sigma-Aldrich, St. Louis, MO). Each plasmid sample was sequenced in both directions with the pJET1.2F (5′-CGACTCACTATAGGGAGAGCGGC-3′) and pJET1.2R (5′-AAGAACATCGATTTTCCATGGCAG-3′) primers by the Sanger method in a commercial facility (ELIM BIOPHARM, Hayward, CA, USA).

#### Bioinformatic analysis

The raw sequences were trimmed to remove the pJET1.2 flanking multiple cloning site sequences with the VecScreen (https://www.ncbi.nlm.nih.gov/tools/vecscreen/). A consensus sequence from each of the sample-specific forward and reverse sequences was derived using the CAP contig assembly program of the BioEdit software^39^. The derived sequences were scanned with the National Center for Biotechnology Information (NCBI) GenBank database using the BLASTN program^40^ for species identification purposes. The MUSCLE alignment program^41^ was used to perform multiple sequence alignments for gene-specific datasets of sequences derived from samples analyzed in this study and corresponding sequences of taxon representative retrieved from GenBank. The gene-specific alignment files were used to determine the sequence identity matrices and for phylogenetic analysis with the maximum likelihood algorithm of the molecular evolutionary genetics analysis (MEGA) software version 7.0^42^.

### Testing for the presence of *Ca.* Liberibacter spp.

DNA extracts from all the ACP samples collected in Benin were assayed for the presence of CLaf, CLam and CLas using the Taqman multiplex real-time PCR assays as reported by Oke et al.^28^. Known positive and negative control DNA samples, and non-template water control were included in the reactions. All samples with a cycle threshold (Ct) ≤ 37 were considered positive for a specific target bacterium.

## Results

### ACP detection and morphological identification

**Figure 1:**
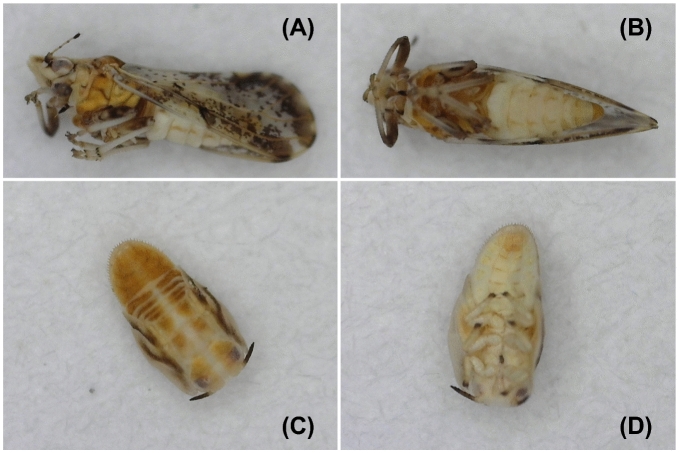
The lateral (**A**) and ventral (**B**) views of adult Asian citrus psyllid (*Diaphorinacitri*Kuwayama) and corresponding dorsal (**C**) and ventral (**D**) views of *D. citri*nymphs sampled from different locations (Table 1) in southern Republic of Benin.

Nine locations (residential sites = 6 and commercial groves = 3) were surveyed during the study (Table 1). Five major ACP host plants including grapefruit, lime, orange jasmine, sweet orange and tangerine were identified at the various locations. Suspect ACP samples were only collected at three residential sites in southern Benin, and none was found in commercial groves and in residential sites in the central and northern departments. The comparison of morphological features of collected samples to voucher specimens^33,36,43^ led to their identification as *D. citri* (Fig. 1). ACP was recovered from three host plants including lime, orange jasmine and sweet orange during the study. While adults were collected from three sites, nymphs were only found on sweet orange trees at one location (Table 1). Considering the spatial separation (2–10 km) of the three detection sites in southern Benin, *D. citri* may likely be established in this part of the country. During the surveys, aphids and soft scales, including the brown soft scale and the cottony cushion scale, were the most dominant pest species found infesting trees, and they are very likely responsible for sooty mold occurring on the trees.

### Molecular detection

A representative subset of 10 randomly collected ACP (4 adults and 6 nymphs) from the three positive detection sites was selected for molecular analysis. Gene-specific DNA amplicons of the expected sizes were obtained from these samples. Ten *mtCOI*-specific (GenBank accession no. OP414612–OP414621), 8 *argH*-specific (OP414446–OP414453) and 10 *atpA*-specific (OP414454–OP414463) sequences were derived. Analysis of these sequences using BLASTN produced highly significant matches (≥ 99% nt identity; 100% query coverage; E-value 0.0) to corresponding gene-specific sequences of *D. citri*, *Ca*. Carsonella ruddii and *Ca*. Profftella armatura, respectively available in GenBank. In pairwise comparisons, the *mtCOI* sequences derived in this study shared 99–100% *nt* identities among themselves and the same range of *nt* identities with corresponding global sequences of *D. citri*, indicating that they belong to this psyllid species. As expected, the derived *mtCOI* sequences from the Benin insect samples were significantly distinct from (and shared only 42–43% identity with) the corresponding sequences of *T. erytreae* isolates retrieved from GenBank. Based on pairwise comparisons, the *argH*- and *atpA*-specific sequences derived in this study shared ~ 100% *nt* identity with their respective gene-specific sequences and 97–100% and 99.5–99.8% *nt* identity with corresponding global sequences of *Ca*. Carsonella ruddii (P-endosymbiont) and *Ca*. Profftella armatura (S-endosymbiont), respectively. The results provide definitive confirmation of the identity of the Benin suspect individuals as *D. citri* and showed that they carry the same primary and secondary bacterial endosymbionts in their bacteriome as previously documented in ACP from other citrus-producing areas of the world^20,44,45^.

### Phylogenetic analysis

**Figure 2:**
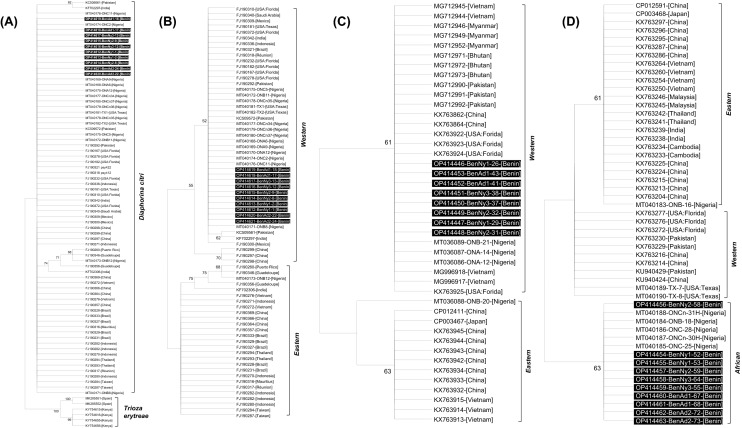
Maximum Likelihood (ML) phylogenetic trees depicting the evolutionary relationships between adults and nymphs of the Asian citrus psyllid (*Diaphorina citri* Kuwayama), and their primary and secondary endosymbionts, sampled from different locations (Table 1) in southern Republic of Benin, West Africa and the corresponding sequences of global populations of each taxon. The ML trees were derived based on analyses of sequences specific to the *mtCOI* gene of *D. citri* (**A** and **B**): OP414612 to OP414621 derived in this study and others from GenBank; the argH gene of the primary endosymbiont *Ca*. Carsonella ruddii (**C**): OP414446 to OP414453 derived in this study and others from GenBank; and the atpA gene of the secondary endosymbiont *Ca*. Profftella armatura (**D**): OP414454 to OP414463 derived in this study and others from GenBank. The sequences derived in this study are shaded in black color. The Tamura 3-parameter was determined as the model with the lowest BIC (Bayesian Information Criterion) scores and was therefore used in ML phylogenetic analysis for each of the gene-specific sequences (with 1000 bootstrap replications). Branches with < 60% bootstrap support were collapsed.

The reconstructed *mtCOI* maximum likelihood (ML) phylogenetic trees showed the clustering of sequences from Benin within the *D. citri* clade and distinct from the *T. erytreae* clade (Fig. 2A). When the ML tree was reconstructed with only *D. citri mtCOI* sequences, they clearly segregated into the previously defined Western and Eastern clades^20^ with strong (> 50%) bootstrap support (Fig. 2B). All the adult and nymph *mtCOI* sequences from Benin (OP414612 to OP414621) clustered into the Western clade (Fig. 2B). The *argH*-specific sequences of the P-endosymbiont *Ca*. Carsonella ruddii also segregated into the previously defined Western and Eastern clades^46^ with strong (> 60%) bootstrap support (Fig. 2C, Supplementary table). Like the ACP *mtCOI* sequences, all the *argH* sequences from Benin (n = 8; GenBank acc. Nos. OP414446–OP414453) also clustered into the Western clade (Fig. 2C). In contrast to the *mtCOI* and *argH* sequences, the *atpA* sequences of the S-endosymbiont *Ca*. Profftella armatura segregated into three distinct clades with strong (> 60%) bootstrap support (Fig. 2D, Supplementary table). Interestingly, all the *atpA* sequences from Benin (n = 10; GenBank acc. Nos. OP414454–OP414463) segregated into the previously defined ‘African’ clade, distinct from the Western and Eastern clades (Fig. 2D). This ‘African’ clade was derived from *atpA* gene of *Ca*. Proftella armatura detected in ACP collected in Nigeria^28^. Taken together, the results showed that the field-collected psyllids in Benin and their bacterial endosymbionts are genetically uniform and belonged to the species *Diaphorina citri*. All the analyzed psyllid samples were negative for CLaf, CLam and CLas by qPCR.

## Discussion

**Figure 3:**
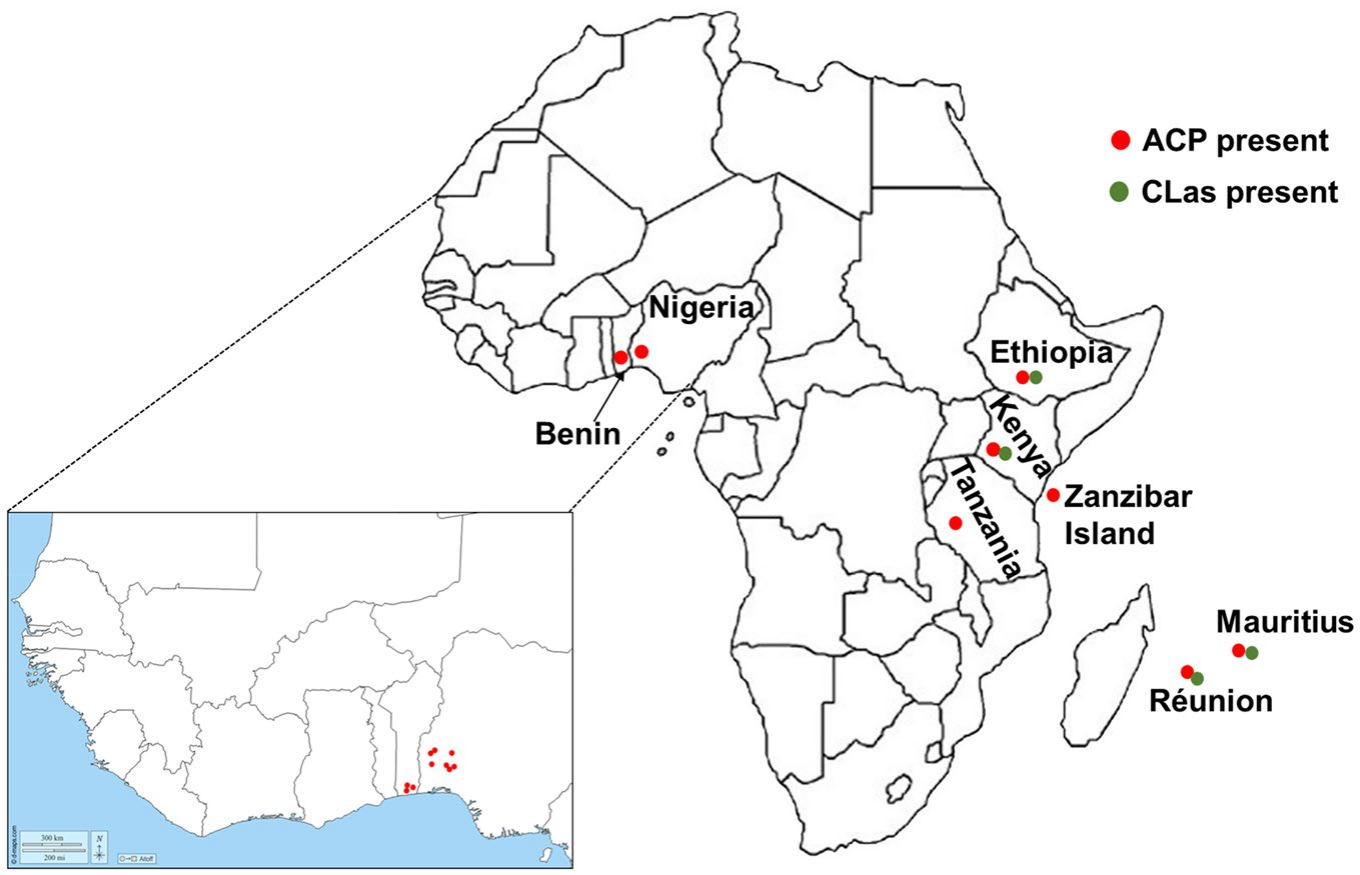
Map of Africa showing current incidence of the *Candidatus* Liberibacterasiaticus (CLas) and its Asian citrus psyllid (ACP) vector. The approximate locations where the ACP was detected in Nigeria (Oke et al.^28^) and the Republic of Benin (this study) are shown in the enlarged map of West Africa.

Using both morphological identification and molecular tools, our study confirmed the suspect specimens collected in Benin to be *D. citri*. While *D. citri* has long been recognized as an invasive pest^22^, this is to the best of our knowledge of current literature, the first report of its detection in Benin. The presence of ACP on the African continent was previously reported from countries in East Africa including Tanzania^21^, Kenya^26^ and Ethiopia^27^, and in Nigeria in West Africa^28^ (Fig. 3). The current detection in Benin shows that the geographic range of ACP is expanding on the continent, possibly through the increased trade among countries, movement of citrus transplants by hobbyist growers, or natural dispersal. However, its presence in only three residential sites in the south suggests that its spread may be currently limited in the country. Surprisingly, the ACP was detected feeding on three of the most suitable host plants^47,48^ in the coastal part of the country with altitude less than 20 m above sea level (Table 1). It is well established that higher altitude limits the incidence of the ACP with its population levels decreasing at higher altitudes probably as a result of differential temperature, air pressure, oxygen level, ultraviolet light or their combinations^49^. However, all sampling sites were below 600 m above sea level, the cut-off point above which no ACP was collected in Puerto Rio^49^. The limited distribution of the ACP in Benin opens avenues for effective management and the enactment of strict quarantine regulations to prevent the incursion of *D. citri* into the commercial citrus groves mostly located in southern and central parts of the country. The prevailing hot and humid conditions in Benin with temperature ranging from 28 to 32 °C all year round, indicate that the whole country is suitable for the establishment and development of *D. citri*. Indeed, using a temperature-based model of suitability, Taylor et al.^50^ reported that Africa has climate suitable for the establishment of *D. citri* and HLB. Considering the biology and ecology of *D. citri*, Aidoo et al.^51^ used Maximum Entropy (MaxEnt) to predict that all citrus production areas in Africa are suitable for its establishment although the actual suitability varies with regions. Under current climatic conditions, Benin lies from the high suitability area in the southern and central parts of the country to the medium suitability area in the north^51^, indicating that the whole country is at high risk of *D. citri* and HLB invasion. The commercial citrus belt is located in the southern part of the country that is deemed highly suitable for *D. citri* reproduction and development and only situated ~ 100 km away from the current detection sites. This close proximity of the commercial citrus production area to the current *D. citri* detection sites represents a grave menace to the sustainability of citriculture in Benin. Citrus production in Benin is already crippled by a myriad of biotic and abiotic constraints and a lack of agrochemical inputs. The invasion of *D. citri* and possible HLB introduction will further reduce yields that are already among the lowest in the world^6^. Hence, a rapid response plan needs to be implemented at the national level, and perhaps regionally across West Africa, to limit the spread of this invasive pest and to control its population within currently established areas.

To gain more insights into the biodiversity of this quarantine significant pest, we analyzed the mitochondrial cytochrome oxidase *(mtCOI*) gene of specimens collected in Benin. Not only that the *mtCOI* is versatile because of its high prevalence in cells relative to nuclear genes*,* it is a highly conserved and maternally inherited gene that is independent of life stages, polymorphism and gender^52,53^. The *mtCOI* gene has been used with great success in previous *D. citri* population studies^20,28,38,54,55^. Similarly, the gene-specific sequence of the primary (*Ca*. Carsonella ruddii) and secondary (*Ca*. Profftella armatura) endosymbionts of *D. citri* were used to evaluate their diversity. All the analyzed *D. citri* individuals from Benin and their primary endosymbiont (*Ca*. Carsonella ruddii) formed a homogenous group and clustered within the Western clade that consists of psyllid populations that generally include individuals from Asia and the Americas^46,56,57^. However, all the *atpA* sequences from Benin segregated into the previously defined ‘African’ clade derived from *D. citri* collected from neighboring Nigeria, and distinct from the Western and Eastern clades^28^. These results highly suggest that the *D. citri* samples collected from Benin and Nigeria may be of the same origin and given the close proximity of both countries (Fig. 3), it is highly plausible that this pest may have spread from one country to another. Moreover, these results points to the fact that *D. citri* may also have been present for quite some time in Benin for local adaptation of the secondary endosymbiont to occur as previously hypothesized^28^. Such local adaptation of *Ca*. Profftella armatura may be warranted due to its role as a defensive endosymbiont against natural enemies in insects^45^.

The presence of sooty mold on foliage and fruit of the surveyed host plants was the precursor of this study. Sooty mold is generally a cosmetic problem on fruit, but its heavy presence as observed on some leaves can impair photosynthesis, leading to poor tree growth and productivity. This is because the black coating of the sooty mold fungi on leaves could intercept sunlight and perturb leaf temperature and transpiration, subsequently affecting the water balance of trees^58^. Although, the ACP was found at one location where sooty mold was recorded on plants, the main culprits of this symptom were soft scales (brown soft scale and cottony cushion scale), aphids and whiteflies that were abundant throughout the country, indicative of poor pest management in citrus production in Benin. In addition, at that site both ACP nymphs and adults were recorded, probably because of the presence of young shoots on sweet orange trees (Table 1). The ACP is known to reproduce exclusively on young shoots^35^ that were absent in most locations at the time of sampling.

Increasing citrus production in Benin will require inputs of agrochemicals, irrigation, and effective locally-adapted integrated pest and disease management (IPDM). The detection of *D. citri* in Benin poses another significant challenge for the sustainability of citrus production, especially if *D. citri* were to acquire *Candidatus* Liberibacter asiaticus, causal agent of HLB. Insights gleaned from other invaded citrus producing areas of the world indicate that HLB detection always (and most often surely) lag *D. citri* detection^18,19,59^. One major foundation for the development of an effective IPDM program will be an understanding of the identity and population dynamics of pests and disease cycles in commercial citrus groves. With the worldwide spread of invasive species, area-wide surveys need to be frequently conducted for early detection of any introduced pest and disease and subsequent implementation of eradication or mitigation approaches.

This work represents a limited and quick study to assess the presence of psyllid vectors of HLB in Benin. The positive detection of *D. citri* following that of neighboring Nigeria in 2020^28^ calls for extensive and regional field surveys to determine the extent of its spread across West Africa and especially in the commercial citrus production areas. Although all the *D. citri* samples analyzed in this study and those from Oke et al.^28^ tested negative for the HLB bacterium, it will be important to continue to systematically survey, sample, and test *D. citri* from the study sites and surrounding areas since CLas detection in psyllids often predates detection in trees^59^. In conclusion, the current restricted distribution of *D. citri* in Benin offers an opportunity for limiting its spread and effectively controlling its populations (“Supplemantary information”).

## Supplementary Information

Supplementary Information.

## Data Availability

The datasets generated and/or analyzed during this study are available in the GenBank. For the mtCOI-specific (GenBank accession no. OP414612–OP414621). SUB12050645 BenNy1-1 OP414612; SUB12050645 BenNy1-2 OP414613; SUB12050645 BenNy2-8 OP414614; SUB12050645 BenNy2-9 OP414615; SUB12050645 BenNy3-12 OP414616; SUB12050645 BenNy3-13OP414617; SUB12050645 BenAd1-17 OP414618; SUB12050645 BenAd1-18 OP414619; SUB12050645 BenAd2-22 OP414620; SUB12050645 BenAd2-24 OP414621. A copy of the files can be viewed at: https://nam04.safelinks.protection.outlook.com/?url=https%3A%2F%2Fsubmit.ncbi.nlm.nih.gov%2Fsubs%2F%3Fsearch%3DSUB12050645&data=05%7C01%7CMamoudou.Setamou%40tamuk.edu%7Cbbbb36a7a1d147a8554a08da9d82ea60%7C17420fd64d7546859adf7a650964dbcf%7C0%7C0%7C637995482851818680%7CUnknown%7CTWFpbGZsb3d8eyJWIjoiMC4wLjAwMDAiLCJQIjoiV2luMzIiLCJBTiI6Ik1haWwiLCJXVCI6Mn0%3D%7C3000%7C%7C%7C&sdata=q96cGgwGIfL%2FU68uEYMON25c%2FxBXUQUH%2Ft3wD2qHQUY%3D&reserved=0. For the argH-specific (OP414446 to OP414453) and the 10 atpA-specific (OP414454 to OP414463) and also herein provided as supplemental materials.
