# Supplementary material for: Report on the first detection of Asian citrus psyllid Diaphorina citri Kuwayama (Hemiptera: Liviidae) in the Republic of Benin, West Africa
Source: Sci Rep. 2023 Jan 16;13:801. doi: 10.1038/s41598-023-28030-3 (PMC9842724; doi:10.1038/s41598-023-28030-3)
Supplement: Supplementary file 1 — Supplementary Information. [file 41598_2023_28030_MOESM1_ESM.docx]

# For Scientific Reports

# Report on the first detection of Asian citrus psyllid *Diaphorina citri* Kuwayama (Hemiptera: Liviidae) in the Republic of Benin, West Africa

Mamoudou Sétamou^1^*, Yovanna L. Soto^1^, Martine Tachin^2^, Olufemi J. Alabi^3^

^1^Texas A&M University-Kingsville Citrus Center, Weslaco 78599

^2^School of Horticulture and Management of Green Spaces, National University of Agriculture (UNA), Kétou, Republic of Benin

^3^Department of Plant Pathology & Microbiology, Texas A&M AgriLife Research & Extension Center, Weslaco, TX 78596

*Corresponding authors: M. Sétamou <[mamoudou.setamou@tamuk.edu](mailto:mamoudou.setamou@tamuk.edu)>

**ACP-mtCOI_Benin**

>BenNy1-1

AGGAGGTGGAGACCCAATCTTATATCAACATTTATTCTGATTTTTTGGACATCCTGAAGTGTACATTCTTATTCTTCCGGGGTTTGGGTTAATCTCACATATTACTACACAAGAAAGAGGTAAAACTTCAGCATTCGGTACATTAGGAATAATTTATGCTATATTAGCTATTGGAATTTTAGGATTTATTGTATGAGCTCACCACATATTTACTGTAGGAATAGATGTAGACTCACGAGCTTATTTTACTTCAGCAACCATAATTATTGCAGTACCGACAGGAATTAAAATTTTTAGTTGACTAGCTACAATTTACGGAATAAAAATAAAATTTTCTCCAAGTATTTCATGATCATTAGGATTTATCTTCCTTTTTACAGTAGGAGGACTTACAGGAGTAATTTTAGCAAATTCATCAATTGACATTATTCTTCATGACACTTATTATGTAGTTGCCCACTTTCACTATGTGCTATCAATAGGGGCTGTATTTGCCATTATTGCTAGATTTATTAATTGATACCCTTTATTAACAGGAAATACAATAAATAAAACTTTACTTAAAGCTCAATTTTTAAGAACCTTCGTCGGAGTAAACACTACCTTTTTCCCCCAACACTTCTTAGGACTTATAGGAATGCCACGACGATACTCGAACTATCCAGATCTTCTCATTTTCTGAAATATTATTTCTTCCTTAGGGTCTATAATTTCACTATTTTCAGTATTACTACTTATAATCATTATCTGAGAAGCAATAACTTCTAATCGAATTGTTTTATTCAATAGAAATTATATAATAATAGAATGAATGCAAAACTCTCCCCCAATTGA

>BenNy1-2

AGGAGGTGGAGACCCAATCTTATATCAACATTTATTCTGATTTTTTGGACATCCTGAAGTGTACATTCTTATTCTTCCGGGGTTTGGGTTAATCTCACATATTACTACACAAGAAAGAGGTAAAACTTCAGCATTCGGTACATTAGGAATAATTTATGCTATATTAGCTATTGGAATTTTAGGATTTATTGTATGAGCTCACCACATATTTACTGTAGGAATAGATGTAGACTCACGAGCTTATTTTACTTCAGCAACCATAATTATTGCAGTACCGACAGGAATTAAAATTTTTAGTTGACTAGCTACAATTTACGGAATAAAAATAAAATTTTCTCCAAGTATTTCATGATCATTAGGATTTATCTTCCTTTTTACAGTAGGAGGACTTACAGGAGTAATTTTAGCAAATTCATCAATTGACATTATTCTTCATGACACTTATTATGTAGTTGCCCACTTTCACTATGTGCTATCAATAGGGGCTGTATTTGCCATTATTGCTAGATTTATTAATTGATACCCTTTATTAACAGGAAATACAATAAATAAAACTTTACTTAAAGCTCAATTTTTAAGAACCTTCGTCGGAGTAAACACTACCTTTTTCCCCCAACACTTCTTAGGACTTATAGGAATGCCACGACGATACTCGAACTATCCAGATCTTCTCATTTTCTGAAATATTATTTCTTCCTTAGGGTCTATAATTTCACTATTTTCAGTATTACTACTTATAATCATTATCTGAGAAGCAATAACTTCTAATCGAATTGTTTTATTCAATAGAAATTATATAATAATAGAATGAATGCAAAACTCTCCCCCAATTGA

>BenNy2-8

AGGAGGTGGAGACCCAATCTTATATCAACATTTATTCTGATTTTTTGGACATCCTGAAGTGTACATTCTTATTCTTCCGGGGTTTGGGTTAATCTCACATATTACTACACAAGAAAGAGGTAAAACTTCAGCATTCGGTACATTAGGAATAATTTATGCTATATTAGCTATTGGAATTTTAGGATTTATTGTATGAGCTCACCACATATTTACTGTAGGAATAGATGTAGACTCACGAGCTTATTTTACTTCAGCAACCATAATTATTGCAGTACCGACAGGAATTAAAATTTTTAGTTGACTAGCTACAATTTACGGAATAAAAATAAAATTTTCTCCAAGTATTTCATGATCATTAGGATTTATCTTCCTTTTTACAGTAGGAGGACTTACAGGAGTAATTTTAGCAAATTCATCAATTGACATTATTCTTCATGACACTTATTATGTAGTTGCCCACTTTCACTATGTGCTATCAATAGGGGCTGTATTTGCCATTATTGCTAGATTTATTAATTGATACCCTTTATTAACAGGAAATACAATAAATAAAACTTTACTTAAAGCTCAATTTTTAAGAACCTTCGTCGGAGTAAACACTACCTTTTTCCCCCAACACTTCTTAGGACTTATAGGAATGCCACGACGATACTCGAACTATCCAGATCTTCTCATTTTCTGAAATATTATTTCTTCCTTAGGGTCTATAATTTCACTATTTTCAGTATTACTACTTATAATCATTATCTGAGAAGCAATAACTTCTAATCGAATTGTTTTATTCAATAGAAATTATATAATAATAGAATGAATGCAAAACTCTCCCCCAATTGA

>BenNy2-9

AGGAGGTGGAGACCCAATCTTATATCAACATTTATTCTGATTTTTTGGACATCCTGAAGTGTACATTCTTATTCTTCCGGGGTTTGGGTTAATCTCACATATTACTACACAAGAAAGAGGTAAAACTTCAGCATTCGGTACATTAGGAATAATTTATGCTATATTAGCTATTGGAATTTTAGGATTTATTGTATGAGCTCACCACATATTTACTGTAGGAATAGATGTAGACTCACGAGCTTATTTTACTTCAGCAACCATAATTATTGCAGTACCGACAGGAATTAAAATTTTTAGTTGACTAGCTACAATTTACGGAATAAAAATAAAATTTTCTCCAAGTATTTCATGATCATTAGGATTTATCTTCCTTTTTACAGTAGGAGGACTTACAGGAGTAATTTTAGCAAATTCATCAATTGACATTATTCTTCATGACACTTATTATGTAGTTGCCCACTTTCACTATGTGCTATCAATAGGGGCTGTATTTGCCATTATTGCTAGATTTATTAATTGATACCCTTTATTAACAGGAAATACAATAAATAAAACTTTACTTAAAGCTCAATTTTTAAGAACCTTCGTCGGAGTAAACACTACCTTTTTCCCCCAACACTTCTTAGGACTTATAGGAATGCCACGACGATACTCGAACTATCCAGATCTTCTCATTTTCTGAAATATTATTTCTTCCTTAGGGTCTATAATTTCACTATTTTCAGTATTACTACTTATAATCATTATCTGAGAAGCAATAACTTCTAATCGAATTGTTTTATTCAATAGAAATTATATAATAATAGAATGAATGCAAAACTCTCCCCCAATTGA

>BenNy3-12

AGGAGGTGGAGACCCAATCTTATATCAACATTTATTCTGATTTTTTGGACATCCTGAAGTGTACATTCTTATTCTTCCGGGGTTTGGGTTAATCTCACATATTACTACACAAGAAAGAGGTAAAACTTCAGCATTCGGTACATTAGGAATAATTTATGCTATATTAGCTATTGGAATTTTAGGATTTATTGTATGAGCTCACCACATATTTACTGTAGGAATAGATGTAGACTCACGAGCTTATTTTACTTCAGCAACCATAATTATTGCAGTACCGACAGGAATTAAAATTTTTAGTTGACTAGCTACAATTTACGGAATAAAAATAAAATTTTCTCCAAGTATTTCATGATCATTAGGATTTATCTTCCTTTTTACAGTAGGAGGACTTACAGGAGTAATTTTAGCAAATTCATCAATTGACATTATTCTTCATGACACTTATTATGTAGTTGCCCACTTTCACTATGTGCTATCAATAGGGGCTGTATTTGCCATTATTGCTAGATTTATTAATTGATACCCTTTATTAACAGGAAATACAATAAATAAAACTTTACTTAAAGCTCAATTTTTAAGAACCTTCGTCGGAGTAAACACTACCTTTTTCCCCCAACACTTCTTAGGACTTATAGGAATGCCACGACGATACTCGAACTATCCAGATCTTCTCATTTTCTGAAATATTATTTCTTCCTTAGGGTCTATAATTTCACTATTTTCAGTATTACTACTTATAATCATTATCTGAGAAGCAATAACTTCTAATCGAATTGTTTTATTCAATAGAAATTATATAATAATAGAATGAATGCAAAACTCTCCCCCAATTG

>BenNy3-13

AGGAGGTGGAGACCCAATCTTATATCAACATTTATTCTGATTTTTTGGACATCCTGAAGTGTACATTCTTATTCTTCCGGGGTTTGGGTTAATCTCACATATTACTACACAAGAAAGAGGTAAAACTTCAGCATTCGGTACATTAGGAATAATTTATGCTATATTAGCTATTGGAATTTTAGGATTTATTGTATGAGCTCACCACATATTTACTGTAGGAATAGATGTAGACTCACGAGCTTATTTTACTTCAGCAACCATAATTATTGCAGTACCGACAGGAATTAAAATTTTTAGTTGACTAGCTACAATTTACGGAATAAAAATAAAATTTTCTCCAAGTATTTCATGATCATTAGGATTTATCTTCCTTTTTACAGTAGGAGGACTTACAGGAGTAATTTTAGCAAATTCATCAATTGACATTATTCTTCATGACACTTATTATGTAGTTGCCCACTTTCACTATGTGCTATCAATAGGGGCTGTATTTGCCATTATTGCTAGATTTATTAATTGATACCCTTTATTAACAGGAAATACAATAAATAAAACTTTACTTAAAGCTCAATTTTTAAGAACCTTCGTCGGAGTAAACACTACCTTTTTCCCCCAACACTTCTTAGGACTTATAGGAATGCCACGACGATACTCGAACTATCCAGATCTTCTCATTTTCTGAAATATTATTTCTTCCTTAGGGTCTATAATTTCACTATTTTCAGTATTACTACTTATAATCATTATCTGAGAAGCAATAACTTCTAATCGAATTGTTTTATTCAATAGAAATTATATAATAATAGAATGAATGCAAAACTCTCCCCCAATTGA

>BenAd1-17

AGGAGGTGGAGACCCAATCTTATATCAACATTTATTCTGATTTTTTGGACATCCTGAAGTGTACATTCTTATTCTTCCGGGGTTTGGGTTAATCTCACATATTACTACACAAGAAAGAGGTAAAACTTCAGCATTCGGTACATTAGGAATAATTTATGCTATATTAGCTATTGGAATTTTAGGATTTATTGTATGAGCTCACCACATATTTACTGTAGGAATAGATGTAGACTCACGAGCTTATTTTACTTCAGCAACCATAATTATTGCAGTACCGACAGGAATTAAAATTTTTAGTTGACTAGCTACAATTTACGGAATAAAAATAAAATTTTCTCCAAGTATTTCATGATCATTAGGATTTATCTTCCTTTTTACAGTAGGAGGACTTACAGGAGTAATTTTAGCAAATTCATCAATTGACATTATTCTTCATGACACTTATTATGTAGTTGCCCACTTTCACTATGTGCTATCAATAGGGGCTGTATTTGCCATTATTGCTAGATTTATTAATTGATACCCTTTATTAACAGGAAATACAATAAATAAAACTTTACTTAAAGCTCAATTTTTAAGAACCTTCGTCGGAGTAAACACTACCTTTTTCCCCCAACACTTCTTAGGACTTATAGGAATGCCACGACGATACTCGAACTATCCAGATCTTCTCATTTTCTGAAATATTATTTCTTCCTTAGGGTCTATAATTTCACTATTTTCAGTATTACTACTTATAATCATTATCTGAGAAGCAATAACTTCTAATCGAATTGTTTTATTCAATAGAAATTATATAATAATAGAATGAATGCAAAACTCTCCCCCAATTGA

>BenAd1-18

AGGAGGTGGAGACCCAATCTTATATCAACATTTATTCTGATTTTTTGGACATCCTGAAGTGTACATTCTTATTCTTCCGGGGTTTGGGTTAATCTCACATATTACTACACAAGAAAGAGGTAAAACTTCAGCATTCGGTACATTAGGAATAATTTATGCTATATTAGCTATTGGAATTTTAGGATTTATTGTATGAGCTCACCACATATTTACTGTAGGAATAGATGTAGACTCACGAGCTTATTTTACTTCAGCAACCATAATTATTGCAGTACCGACAGGAATTAAAATTTTTAGTTGACTAGCTACAATTTACGGAATAAAAATAAAATTTTCTCCAAGTATTTCATGATCATTAGGATTTATCTTCCTTTTTACAGTAGGAGGACTTACAGGAGTAATTTTAGCAAATTCATCAATTGACATTATTCTTCATGACACTTATTATGTAGTTGCCCACTTTCACTATGTGCTATCAATAGGGGCTGTATTTGCCATTATTGCTAGATTTATTAATTGATACCCTTTATTAACAGGAAATACAATAAATAAAACTTTACTTAAAGCTCAATTTTTAAGAACCTTCGTCGGAGTAAACACTACCTTTTTCCCCCAACACTTCTTAGGACTTATAGGAATGCCACGACGATACTCGAACTATCCAGATCTTCTCATTTTCTGAAATATTATTTCTTCCTTAGGGTCTATAATTTCACTATTTTCAGTATTACTACTTATAATCATTATCTGAGAAGCAATAACTTCTAATCGAATTGTTTTATTCAATAGAAATTATATAATAATAGAATGAATGCAAAACTCTCCCCCAATTGA

>BenAd2-22

AGGAGGTGGAGACCCAATCTTATACCAACATTTATTCTGATTTTTTGGACATCCTGAAGTGTACATTCTTATTCTTCCGGGGTTTGGGTTAATCTCACATATTACTACACAAGAAAGAGGTAAAACTTCAGCATTCGGTACATTAGGAATAATTTATGCTATATTAGCTATTGGAATTTTAGGATTTATTGTATGAGCTCACCACATATTTACTGTAGGAATAGATGTAGACTCACGAGCTTATTTTACTTCAGCAACCATAATTATTGCAGTACCGACAGGAATTAAAATTTTTAGTTGACTAGCTACAATTTACGGAATAAAAATAAAATTTTCTCCAAGTATTTCATGATCATTAGGATTTATCTTCCTTTTTACAGTAGGAGGACTTACAGGAGTAATTTTAGCAAATTCATCAATTGACATTATTCTTCATGACACTTATTATGTAGTTGCCCACTTTCACTATGTGCTATCAATAGGGGCTGTATTTGCCATTATTGCTAGATTTATTAATTGATACCCTTTATTAACAGGAAATACAATAAATAAAACTTTACTTAAAGCTCAATTTTTAAGAACCTTCGTCGGAGTAAACACTACCTTTTTCCCCCAACACTTCTTAGGACTTATAGGAATGCCACGACGATACTCGAACTATCCAGATCTTCTCATTTTCTGAAATATTATTTCTTCCTTAGGGTCTATAATTTCACTATTTTCAGTATTACTACTTATAATCATTATCTGAGAAGCAATAACTTCTAATCGAATTGTTTTATTCAATAGAAATTATATAATAATAGAATGAATGCAAAACTCTCCCCCAATTGA

>BenAd2-24

AGGAGGTGGAGACCCAATCTTATACCAACATTTATTCTGATTTTTTGGACATCCTGAAGTGTACATTCTTATTCTTCCGGGGTTTGGGTTAATCTCACATATTACTACACAAGAAAGAGGTAAAACTTCAGCATTCGGTACATTAGGAATAATTTATGCTATATTAGCTATTGGAATTTTAGGATTTATTGTATGAGCTCACCACATATTTACTGTAGGAATAGATGTAGACTCACGAGCTTATTTTACTTCAGCAACCATAATTATTGCAGTACCGACAGGAATTAAAATTTTTAGTTGACTAGCTACAATTTACGGAATAAAAATAAAATTTTCTCCAAGTATTTCATGATCATTAGGATTTATCTTCCTTTTTACAGTAGGAGGACTTACAGGAGTAATTTTAGCAAATTCATCAATTGACATTATTCTTCATGACACTTATTATGTAGTTGCCCACTTTCACTATGTGCTATCAATAGGGGCTGTATTTGCCATTATTGCTAGATTTATTAATTGATACCCTTTATTAACAGGAAATACAATAAATAAAACTTTACTTAAAGCTCAATTTTTAAGAACCTTCGTCGGAGTAAACACTACCTTTTTCCCCCAACACTTCTTAGGACTTATAGGAATGCCACGACGATACTCGAACTATCCAGATCTTCTCATTTTCTGAAATATTATTTCTTCCTTAGGGTCTATAATTTCACTATTTTCAGTATTACTACTTATAATCATTATCTGAGAAGCAATAACTTCTAATCGAATTGTTTTATTCAATAGAAATTATATAATAATAGAATGAATGCAAAACTCTCCCCCAATTGA

**argH-Benin**

>BenNy1-26

CTCCTATGCCTGGATTTACTCATTTGCAAATTGCTCAACCAATAACAATAGGACATTATCTATTATCTTGGAATGAAATGTTAAAAAGAGACCATTTAAATTTACTAAATTGCAAAAAAAGTTTACAATATCGCCCTTTAGGATCTGCAGCTTTATCAGGACATAATTACAATATTAATAGAAATACTATTAAAAAATTTTTAAATTTTAAAAATTTAACAGAAAATTCTGTAGATGCAGTAAGCGATAGAGATTATATTGTTATGTTTGCTCATTTTTGTAATTTAATTATTACTCATTTATCTAGAATATCTGAAGATATGATAATTTGGAGTAATAATAACTTTGATTTTTTAAAATTATCAGATTTGATTTCTTCTGGTTCTTCAATAATGCCTCAAAAAAAAAATCCTGATTTATTTGAATTAATAAGAGCAAAAACAGGAAGAATATATGGAAATTCTTTAAGTATTTTAACTATTTTAAAAGCTCAACCGTTGTCATATAATAAAGATAATCAAGAAGACAAAGAAAGTCTCTTCGATAATGTTTATACAATTAAAAAAACTCTTAATTCTTTTAGAAAATGCTTACCAATTTTAAAATTTAATAAAAAAAACATGTATTTTTCGGCTTTGAAAAATTATTCAACAGCAACAGACATGGCTGATTATTTAGTAAAAAAAGGTGTTTTATTTCGAGAAGCACACAAAATAGTAGGTAATTGTATTCAATATTGTGAAAAAAATAATATTAATTTGTTTAATATTTCTTTAAACGAATTAAAAAGATTTAGTAATTTATTTGAAAAAAATATTTTTTATGATTTATCAATTGAAGGATCAATAAAAAATAAAAATATTTACGGAGGTACAGCGCCTAATCAA

>BenNy1-29

CTCCTATGCCTGGATTTACTCATTTGCAAATTGCTCAACCAATAACAATAGGACATTATCTATTATCTTGGAATGAAATGTTAAAAAGAGACCATTTAAATTTACTAAATTGCAAAAAAAGTTTACAATATTGCCCTTTAGGATCTGCAGCTTTATCAGGACATAATTACAATATTAATAGAAATACTATTAAAAAATTTTTAAATTTTAAAAATTTAACAGAAAATTCTGTAGATGCAGTAAGCGATAGAGATTATATTGTTATGTTTGCTCATTTTTGTAATTTAATTATTACTCATTTATCTAGAATATCTGAAGATATGATAATTTGGAGTAATAATAACTTTGATTTTTTAAAATTATCAGATTTGATTTCTTCTGGTTCTTCAATAATGCCTCAAAAAAAAAATCCTGATTTATTTGAATTAATAAGAGCAAAAACAGGAAGAATATATGGAAATTCTTTAAGTATTTTAACTATTTTAAAAGCTCAACCGTTGTCATATAATAAAGATAATCAAGAAGACAAAGAAAGTCTCTTCGATAATGTTTATACAATTAAAAAAACTCTTAATTCTTTTAGAAAATGCTTACCAATTTTAAAATTTAATAAAAAAAACATGTATTTTTCGGCTTTGAAAAATTATTCAACAGCAACAGACATGGCTGATTATTTAGTAAAAAAAGGTGTTTTATTTCGAGAAGCACACAAAATAGTAGGTAATTGTATTCAATATTGTGAAAAAAATAATATTAATTTGTTTAATATTTCTTTAAACGAATTAAAAAGATTTAGTAATTTATTTGAAAAAAATATTTTTTATGATTTATCAATTGAAGGATCAATAAAAAATAAAAATATTTACGGAGGTACAGCGCCTAATCAA

>BenNy2-31

CTCCTATGCCTGGATTTACTCATTTGCAAATTGCTCANCCAATAACAATAGGACATTATCTATTATCTTGGAATGAAATGTTAAAAAGAGACCATTTAAATTTACTAAATTGCAAAAAAAGTTTACAATATTGCCCTTTAGGATCTGCAGCTTTATCAGGACATAATTACAATATTAATAGAAATACTATTAAAAAATTTTTAAATTTTAAAAATTTAACAGAAAATTCTGTAGATGCAGTAAGCGATAGAGATTATATTGTTATGTTTGCTCATTTTTGTAATTTAATTATTACTCATTTATCTAGAATATCTGAAGATATGATAATTTGGAGTAATAATAACTTTGATTTTTTAAAATTATCAGATTTGATTTCTTCTGGTTCTTCAATAATGCCTCAAAAAAAAAATCCTGATTTATTTGAATTAATAAGAGCAAAAACAGGAAGAATATATGGAAATTCTTTAAGTATTTTAACTATTTTAAAAGCTCAACCGTTGTCATATAATAAAGATAATCAAGAAGACAAAGAAAGTCTCTTCGATAATGTTTATACAATTAAAAAAACTCTTAATTCTTTTAGAAAATGCTTACCAATTTTAAAATTTAATAAAAAAAACATGTATTTTTCGGCTTTGAAAAATTATTCAACAGCAACAGACATGGCTGATTATTTAGTAAAAAAAGGTGTTTTATTTCGAGAAGCACACAAAATAGTAGGTAATTGTATTCAATATTGTGAAAAAAATAATATTAATTTGTTTAATATTTCTTTAAACGAATTAAAAAGATTTAGTAATTTATTTGAAAAAAATATTTTTTATGATTTATCAATTGAAGGATCAATAAAAAATAAAAATATTTACGGAGGTACAGCGCCTAATCAA

>BenNy2-32

CTCCTATGCCTGGATTTACTCATTTGCAAATTGCTCAACCAATAACAATAGGACATTATCTATTATCTTGGAATGAAATGTTAAAAAGAGACCATTTAAATTTACTAAATTGCAAAAAAAGTTTACAATATTGCCCTTTAGGATCTGCAGCTTTATCAGGACATAATTACAATATTAATAGAAATACTATTAAAAAATTTTTAAATTTTAAAAATTTAACAGAAAATTCTGTAGATGCAGTAAGCGATAGAGATTATATTGTTATGTTTGCTCATTTTTGTAATTTAATTATTACTCATTTATCTAGAATATCTGAAGATATGATAATTTGGAGTAATAATAACTTTGATTTTTTAAAATTATCAGATTTGATTTCTTCTGGTTCTTCAATAATGCCTCAAAAAAAAAATCCTGATTTATTTGAATTAATAAGAGCAAAAACAGGAAGAATATATGGAAATTCTTTAAGTATTTTAACTATTTTAAAAGCTCAACCGTTGTCATATAATAAAGATAATCAAGAAGACAAAGAAAGTCTCTTCGATAATGTTTATACAATTAAAAAAACTCTTAATTCTTTTAGAAAATGCTTACCAATTTTAAAATTTAATAAAAAAAACATGTATTTTTCGGCTTTGAAAAATTATTCAACAGCAACAGACATGGCTGATTATTTAGTAAAAAAAGGTGTTTTATTTCGAGAAGCACACAAAATAGTAGGTAATTGTATTCAATATTGTGAAAAAAATAATATTAATTTGTTTAATATTTCTTTAAACGAATTAAAAAGATTTAGTAATTTATTTGAAAAAAATATTTTTTATGATTTATCAATTGAAGGATCAATAAAAAATAAAAATATTTACGGAGGTACAGCGCCTAATCAA

>BenNy3-37

CTCCTATGCCTGGATTTACTCATTTGCAAATTGCTCAACCAATAACAATAGGACATTATCTATTATCTTGGAATGAAATGTTAAAAAGAGACCATTTAAATTTACTAAATTGCAAAAAAAGTTTACAATATTGCCCTTTAGGATCTGCAGCTTTATCAGGACATAATTACAATATTAATAGAAATACTATTAAAAAATTTTTAAATTTTAAAAATTTAACAGAAAATTCTGTAGATGCAGTAAGCGATAGAGATTATATTGTTATGTTTGCTCATTTTTGTAATTTAATTATTACTCATTTATCTAGAATATCTGAAGATATGATAATTTGGAGTAATAATAACTTTGATTTTTTAAAATTATCAGATTTGATTTCTTCTGGTTCTTCAATAATGCCTCAAAAAAAAAATCCTGATTTATTTGAATTAATAAGAGCAAAAACAGGAAGAATATATGGAAATTCTTTAAGTATTTTAACTATTTTAAAAGCTCAACCGTTGTCATATAATAAAGATAATCAAGAAGACAAAGAAAGTCTCTTCGATAATGTTTATACAATTAAAAAAACTCTTAATTCTTTTAGAAAATGCTTACCAATTTTAAAATTTAATAAAAAAAACATGTATTTTTCGGCTTTGAAAAATTATTCAACAGCAACAGACATGGCTGATTATTTAGTAAAAAAAGGTGTTTTATTTCGAGAAGCACACAAAATAGTAGGTAATTGTATTCAATATTGTGAAAAAAATAATATTAATTTGTTTAATATTTCTTTAAACGAATTAAAAAGATTTAGTAATTTATTTGAAAAAAATATTTTTTATGATTTATCAATTGAAGGATCAATAAAAAATAAAAATATTTACGGAGGTACAGCGCCTAATCAA

>BenNy3-38

CTCCTATGCCTGGATTTACTCATTTGCAAATTGCTCAACCAATAACAATAGGACATTATCTATTATCTTGGAATGAAATGTTAAAAAGAGACCATTTAAATTTACTAAATTGCAAAAAAAGTTTACAATATTGCCCTTTAGGATCTGCAGCTTTATCAGGACATAATTACAATATTAATAGAAATACTATTAAAAAATTTTTAAATTTTAAAAATTTAACAGAAAATTCTGTAGATGCAGTAAGCGATAGAGATTATATTGTTATGTTTGCTCATTTTTGTAATTTAATTATTACTCATTTATCTAGAATATCTGAAGATATGATAATTTGGAGTAATAATAACTTTGATTTTTTAAAATTATCAGATTTGATTTCTTCTGGTTCTTCAATAATGCCTCAAAAAAAAAATCCTGATTTATTTGAATTAATAAGAGCAAAAACAGGAAGAATATATGGAAATTCTTTAAGTATTTTAACTATTTTAAAAGCTCAACCGTTGTCATATAATAAAGATAATCAAGAAGACAAAGAAAGTCTCTTCGATAATGTTTATACAATTAAAAAAACTCTTAATTCTTTTAGAAAATGCTTACCAATTTTAAAATTTAATAAAAAAAACATGTATTTTTCGGCTTTGAAAAATTATTCAACAGCAACAGACATGGCTGATTATTTAGTAAAAAAAGGTGTTTTATTTCGAGAAGCACACAAAATAGTAGGTAATTGTATTCAATATTGTGAAAAAAATAATATTAATTTGTTTAATATTTCTTTAAACGAATTAAAAAGATTTAGTAATTTATTTGAAAAAAATATTTTTTATGATTTATCAATTGAAGGATCAATAAAAAATAAAAATATTTACGGAGGTACAGCGCCTAATCAA

>BenAd1-41

CTCCTATGCCTGGATTTACTCATTTGCAAATTGCTCAACCAATAACAATAGGACATTATCTATTATCTTGGAATGAAATGTTAAAAAGAGACCATTTAAATTTACTAAATTGCAAAAAAAGTTTACAATATTGCCCTTTAGGATCTGCAGCTTTATCAGGACATAATTACAATATTAATAGAAATACTATTAAAAAATTTTTAAATTTTAAAAATTTAACAGAAAATTCTGTAGATGCAGTAAGCGATAGAGATTATATTGTTATGTTTGCTCATTTTTGTAATTTAATTATTACTCATTTATCTAGAATATCTGAAGATATGATAATTTGGAGTAATAATAACTTTGATTTTTTAAAATTATCAGATTTGATTTCTTCTGGTTCTTCAATAATGCCTCAAAAAAAAAATCCTGATTTATTTGAATTAATAAGAGCAAAAACAGGAAGAATATATGGAAATTCTTTAAGTATTTTAACTATTTTAAAAGCTCAACCGTTGTCATATAATAAAGATAATCAAGAAGACAAAGAAAGTCTCTTCGATAATGTTTATACAATTAAAAAAACTCTTAATTCTTTTAGAAAATGCTTACCAATTTTAAAATTTAATAAAAAAAACATGTATTTTTCGGCTTTGAAAAATTATTCAACAGCAACAGACATGGCTGATTATTTAGTAAAAAAAGGTGTTTTATTTCGAGAAGCACACAAAATAGTAGGTAATTGTATTCAATATTGTGAAAAAAATAATATTAATTTGTTTAATATTTCTTTAAACGAATTAAAAAGATTTAGTAATTTATTTGAAAAAAATATTTTTTATGATTTATCAATTGAAGGATCAATAAAAAATAAAAATATTTACGGAGGTACAGCGCCTAATCAA

>BenAd1-43

CTCCTATGCCTGGATTTACTCATTTGCAAATTGCTCAACCAATAACAATAGGACATTATCTATTATCTTGGAATGAAATGTTAAAAAGAGACCATTTAAATTTACTAAATTGCAAAAAAAGTTTACAATATTGCCCTTTAGGATCTGCAGCTTTATCAGGACATAATTACAATATTAATAGAAATACTATTAAAAAATTTTTAAATTTTAAAAATTTAACAGAAAATTCTGTAGATGCAGTAAGCGATAGAGATTATATTGTTATGTTTGCTCATTTTTGTAATTTAATTATTACTCATTTATCTAGAATATCTGAAGATATGATAATTTGGAGTAATAATAACTTTGATTTTTTAAAATTATCAGATTTGATTTCTTCTGGTTCTTCAATAATGCCTCAAAAAAAAAATCCTGATTTATTTGAATTAATAAGAGCAAAAACAGGAAGAATATATGGAAATTCTTTAAGTATTTTAACTATTTTAAAAGCTCAACCGTTGTCATATAATAAAGATAATCAAGAAGACAAAGAAAGTCTCTTCGATAATGTTTATACAATTAAAAAAACTCTTAATTCTTTTAGAAAATGCTTACCAATTTTAAAATTTAATAAAAAAAACATGTATTTTTCGGCTTTGAAAAATTATTCAACAGCAACAGACATGGCTGATTATTTAGTAAAAAAAGGTGTTTTATTTCGAGAAGCACACAAAATAGTAGGTAATTGTATTCAATATTGTGAAAAAAATAATATTAATTTGTTTAATATTTCTTTAAACGAATTAAAAAGATTTAGTAATTTATTTGAAAAAAATATTTTTTATGATTTATCAATTGAAGGATCAATAAAAAATAAAAATATTTACGGAGGTACAGCGCCTAATCAA

**atpA_Benin**

>BenNy1-52

CAATAATCGGTATCGCTGTTAAAGATCCAGTTTTACCAGTTACTTTACCGTTTGTATAATTTTCTACATATTTAATATTTACTCTAGCTGAGCGTTCTAATAATCGACTATGTAAATAAAATATATCCCCAGGATAAGCCTCTCTTCCTGGTGGTCGTTTTAAGAGTAATGATATTTGACGATAAGCAACAGCTTGTTTAGATAAATCGTCATAAACAACTAATGCATCTTTTCCATGATCTCTAAAATATTCTCCAATAGTACAACCTGAATAAGCTGATATAAATTGCATAGAAGCAGAATCAGAAGCTGTTGCAGCTACAATAATAGTATATGGCATTGCTCCATATTTTTCTAATAAATTTGCGGTTTTTTTAATTGATGATATCTTTTGTCCAATAGCAACATAAATACAAGTAACATTTTTATTTTTTTGATTAATAATTATGTCTATTGCTATGGAAGATTTTCCAGTTTGTCTATCTCCAATAATTAATTCACGTTGACCTTTTCCAATTGGCACTATAGAATCTATTGCTTTAATACCTGTTTGCAGGGGTTCGGAGACTGATTGTCGAGAAATTACTCCGGGAGCAACTTTTTCAATTGGAGCAGTTAATTTTGTTTTAATTGGCCCTTTTCCGTCAATTGGATCACCAAGAGCATTAATGACTCGACCACATAATTCCGGGCCAATAGGAACTTCTAAAATACGTCCTGTACATTTAACTATATCACCTTCCGTAATATGCT

>BenNy1-53

CAATAATCGGTATCGCTGTTAAAGATCCAGTTTTACCAGTTACTTTACCGTTTGTATAATTTTCTACATATTTAATATTTACTCTAGCTGAGCGTTCTAATAATCGACTATGTAAATAAAATATATCCCCAGGATAAGCCTCTCTTCCTGGTGGTCGTTTTAAGAGTAATGATATTTGACGATAAGCAACAGCTTGTTTAGATAAATCGTCATAAACAACTAATGCATCTTTTCCATGATCTCTAAAATATTCTCCAATAGTACAACCTGAATAAGCTGATATAAATTGCATAGAAGCAGAATCAGAAGCTGTTGCAGCTACAATAATAGTATATGGCATTGCTCCATATTTTTCTAATAAATTTGCGGTTTTTTTAATTGATGATATCTTTTGTCCAATAGCAACATAAATACAAGTAACATTTTTATTTTTTTGATTAATAATTATGTCTATTGCTATGGAAGATTTTCCAGTTTGTCTATCTCCAATAATTAATTCACGTTGACCTTTTCCAATTGGCACTATAGAATCTATTGCTTTAATACCTGTTTGCAGGGGTTCGGAGACTGATTGTCGAGAAATTACTCCGGGAGCAACTTTTTCAATTGGAGCAGTTAATTTTGTTTTAATTGGCCCTTTTCCGTCAATTGGATCACCAAGAGCATTAATGACTCGACCACATAATTCCGGGCCAATAGGAACTTCTAAAATACGTCCTGTACATTTAACTATATCACCTTCCGTAATATGCT

>BenNy2-58

CAATAATCGGTATCGCTGTTAAAGATCCAGTTTTACCAGTTACTTTACCGTTTGTATAATTTTCTACATATTTAATATTTACTCTAGCTGAGCGTTCTAATAATCGACTATGTAAATAAAATATATCCCCAGGATAAGCCTCTCTTCCTGGTGGTCGTTTTAAGAGTAATGATATTTGACGATAAGCAACAGCTTGTTTAGATAAATCGTCATAAACAACTAATGCATCTTTTCCATGATCTCTAAAATATTCTCCAATAGTACAACCTGAATAAGCTGATATAAATTGCATAGAAGCAGAATCAGAAGCTGTTGCAGCTACAATAATAGTATATGGCATTGCTCCATATTTTTCTAATAAATTTGCGGTTTTTTTAATTGATGATATCTTTTGTCCAATAGCAACATAAATACAAGTAACATTTTTATTTTTTTGATTAATAATTATGTCTATTGCTATGGAAGATTTTCCAGTTTGTCTATCTCCAATAATTAATTCACGTTGACCCTTTCCAATTGGCACTATAGAATCTATTGCTTTAATACCTGTTTGCAGGGGTTCGGAGACTGATTGTCGAGAAATTACTCCGGGAGCAACTTTTTCAATTGGAGCAGTTAATTTTGTTTTAATTGGCCCTTTTCCGTCAATTGGATCACCAAGAGCATTAATGACTCGACCACATAATTCCGGGCCAATAGGAACTTCTAAAATACGTCCTGTACATTTAACTATATCACCTTCCGTAATATGCT

>BenNy2-59

CAATAATCGGTATCGCTGTTAAAGATCCAGTTTTACCAGTTACTTTACCGTTTGTATAATTTTCTACATATTTAATATTTACTCTAGCTGAGCGTTCTAATAATCGACTATGTAAATAAAATATATCCCCAGGATAAGCCTCTCTTCCTGGTGGTCGTTTTAAGAGTAATGATATTTGACGATAAGCAACAGCTTGTTTAGATAAATCGTCATAAACAACTAATGCATCTTTTCCATGATCTCTAAAATATTCTCCAATAGTACAACCTGAATAAGCTGATATAAATTGCATAGAAGCAGAATCAGAAGCTGTTGCAGCTACAATAATAGTATATGGCATTGCTCCATATTTTTCTAATAAATTTGCGGTTTTTTTAATTGATGATATCTTTTGTCCAATAGCAACATAAATACAAGTAACATTTTTATTTTTTTGATTAATAATTATGTCTATTGCTATGGAAGATTTTCCAGTTTGTCTATCTCCAATAATTAATTCACGTTGACCTTTTCCAATTGGCACTATAGAATCTATTGCTTTAATACCTGTTTGCAGGGGTTCGGAGACTGATTGTCGAGAAATTACTCCGGGAGCAACTTTTTCAATTGGAGCAGTTAATTTTGTTTTAATTGGCCCTTTTCCGTCAATTGGATCACCAAGAGCATTAATGACTCGACCACATAATTCCGGGCCAATAGGAACTTCTAAAATACGTCCTGTACATTTAACTATATCACCTTCCGTAATATGCT

>BenNy3-64

CAATAATCGGTATCGCTGTTAAAGATCCAGTTTTACCAGTTACTTTACCGTTTGTATAATTTTCTACATATTTAATATTTACTCTAGCTGAGCGTTCTAATAATCGACTATGTAAATAAAATATATCCCCAGGATAAGCCTCTCTTCCTGGTGGTCGTTTTAAGAGTAATGATATTTGACGATAAGCAACAGCTTGTTTAGATAAATCGTCATAAACAACTAATGCATCTTTTCCATGATCTCTAAAATATTCTCCAATAGTACAACCTGAATAAGCTGATATAAATTGCATAGAAGCAGAATCAGAAGCTGTTGCAGCTACAATAATAGTATATGGCATTGCTCCATATTTTTCTAATAAATTTGCGGTTTTTTTAATTGATGATATCTTTTGTCCAATAGCAACATAAATACAAGTAACATTTTTATTTTTTTGATTAATAATTATGTCTATTGCTATGGAAGATTTTCCAGTTTGTCTATCTCCAATAATTAATTCACGTTGACCTTTTCCAATTGGCACTATAGAATCTATTGCTTTAATACCTGTTTGCAGGGGTTCGGAGACTGATTGTCGAGAAATTACTCCGGGAGCAACTTTTTCAATTGGAGCAGTTAATTTTGTTTTAATTGGCCCTTTTCCGTCAATTGGATCACCAAGAGCATTAATGACTCGACCACATAATTCCGGGCCAATAGGAACTTCTAAAATACGTCCTGTACATTTAACTATATCACCTTCCGTAATATGCT

>BenNy3-65

CAATAATCGGTATCGCTGTTAAAGATCCAGTTTTACCAGTTACTTTACCGTTTGTATAATTTTCTACATATTTAATATTTACTCTAGCTGAGCGTTCTAATAATCGACTATGTAAATAAAATATATCCCCAGGATAAGCCTCTCTTCCTGGTGGTCGTTTTAAGAGTAATGATATTTGACGATAAGCAACAGCTTGTTTAGATAAATCGTCATAAACAACTAATGCATCTTTTCCATGATCTCTAAAATATTCTCCAATAGTACAACCTGAATAAGCTGATATAAATTGCATAGAAGCAGAATCAGAAGCTGTTGCAGCTACAATAATAGTATATGGCATTGCTCCATATTTTTCTAATAAATTTGCGGTTTTTTTAATTGATGATATCTTTTGTCCAATAGCAACATAAATACAAGTAACATTTTTATTTTTTTGATTAATAATTATGTCTATTGCTATGGAAGATTTTCCAGTTTGTCTATCTCCAATAATTAATTCACGTTGACCTTTTCCAATTGGCACTATAGAATCTATTGCTTTAATACCTGTTTGCAGGGGTTCGGAGACTGATTGTCGAGAAATTACTCCGGGAGCAACTTTTTCAATTGGAGCAGTTAATTTTGTTTTAATTGGCCCTTTTCCGTCAATTGGATCACCAAGAGCATTAATGACTCGACCACATAATTCCGGGCCAATAGGAACTTCTAAAATACGTCCTGTACATTTAACTATATCACCTTCCGTAATATGCT

>BenAd1-67

CAATAATCGGTATCGCTGTTAAAGATCCAGTTTTACCAGTTACTTTACCGTTTGTATAATTTTCTACATATTTAATATTTACTCTAGCTGAGCGTTCTAATAATCGACTATGTAAATAAAATATATCCCCAGGATAAGCCTCTCTTCCTGGTGGTCGTTTTAAGAGTAATGATATTTGACGATAAGCAACAGCTTGTTTAGATAAATCGTCATAAACAACTAATGCATCTTTTCCATGATCTCTAAAATATTCTCCAATAGTACAACCTGAATAAGCTGATATAAATTGCATAGAAGCAGAATCAGAAGCTGTTGCAGCTACAATAATAGTATATGGCATTGCTCCATATTTTTCTAATAAATTTGCGGTTTTTTTAATTGATGATATCTTTTGTCCAATAGCAACATAAATACAAGTAACATTTTTATTTTTTTGATTAATAATTATGTCTATTGCTATGGAAGATTTTCCAGTTTGTCTATCTCCAATAATTAATTCACGTTGACCTTTTCCAATTGGCACTATAGAATCTATTGCTTTAATACCTGTTTGCAGGGGTTCGGAGACTGATTGTCGAGAAATTACTCCGGGAGCAACTTTTTCAATTGGAGCAGTTAATTTTGTTTTAATTGGCCCTTTTCCGTCAATTGGATCACCAAGAGCATTAATGACTCGACCACATAATTCCGGGCCAATAGGAACTTCTAAAATACGTCCTGTACATTTAACTATATCACCTTCCGTAATATGCT

>BenAd1-68

CAATAATCGGTATCGCTGTTAAAGATCCAGTTTTACCAGTTACTTTACCGTTTGTATAATTTTCTACATATTTAATATTTACTCTAGCTGAGCGTTCTAATAATCGACTATGTAAATAAAATATATCCCCAGGATAAGCCTCTCTTCCTGGTGGTCGTTTTAAGAGTAATGATATTTGACGATAAGCAACAGCTTGTTTAGATAAATCGTCATAAACAACTAATGCATCTTTTCCATGATCTCTAAAATATTCTCCAATAGTACAACCTGAATAAGCTGATATAAATTGCATAGAAGCAGAATCAGAAGCTGTTGCAGCTACAATAATAGTATATGGCATTGCTCCATATTTTTCTAATAAATTTGCGGTTTTTTTAATTGATGATATCTTTTGTCCAATAGCAACATAAATACAAGTAACATTTTTATTTTTTTGATTAATAATTATGTCTATTGCTATGGAAGATTTTCCAGTTTGTCTATCTCCAATAATTAATTCACGTTGACCTTTTCCAATTGGCACTATAGAATCTATTGCTTTAATACCTGTTTGCAGGGGTTCGGAGACTGATTGTCGAGAAATTACTCCGGGAGCAACTTTTTCAATTGGAGCAGTTAATTTTGTTTTAATTGGCCCTTTTCCGTCAATTGGATCACCAAGAGCATTAATGACTCGACCACATAATTCCGGGCCAATAGGAACTTCTAAAATACGTCCTGTACATTTAACTATATCACCTTCCGTAATATGCT

>BenAd2-72

CAATAATCGGTATCGCTGTTAAAGATCCAGTTTTACCAGTTACTTTACCGTTTGTATAATTTTCTACATATTTAATATTTACTCTAGCTGAGCGTTCTAATAATCGACTATGTAAATAAAATATATCCCCAGGATAAGCCTCTCTTCCTGGTGGTCGTTTTAAGAGTAATGATATTTGACGATAAGCAACAGCTTGTTTAGATAAATCGTCATAAACAACTAATGCATCTTTTCCATGATCTCTAAAATATTCTCCAATAGTACAACCTGAATAAGCTGATATAAATTGCATAGAAGCAGAATCAGAAGCTGTTGCAGCTACAATAATAGTATATGGCATTGCTCCATATTTTTCTAATAAATTTGCGGTTTTTTTAATTGATGATATCTTTTGTCCAATAGCAACATAAATACAAGTAACATTTTTATTTTTTTGATTAATAATTATGTCTATTGCTATGGAAGATTTTCCAGTTTGTCTATCTCCAATAATTAATTCACGTTGACCTTTTCCAATTGGCACTATAGAATCTATTGCTTTAATACCTGTTTGCAGGGGTTCGGAGACTGATTGTCGAGAAATTACTCCGGGAGCAACTTTTTCAATTGGAGCAGTTAATTTTGTTTTAATTGGCCCTTTTCCGTCAATTGGATCACCAAGAGCATTAATGACTCGACCACATAATTCCGGGCCAATAGGAACTTCTAAAATACGTCCTGTACATTTAACTATATCACCTTCCGTAATATGCT

>BenAd2-73

CAATAATCGGTATCGCTGTTAAAGATCCAGTTTTACCAGTTACTTTACCGTTTGTATAATTTTCTACATATTTAATATTTACTCTAGCTGAGCGTTCTAATAATCGACTATGTAAATAAAATATATCCCCAGGATAAGCCTCTCTTCCTGGTGGTCGTTTTAAGAGTAATGATATTTGACGATAAGCAACAGCTTGTTTAGATAAATCGTCATAAACAACTAATGCATCTTTTCCATGATCTCTAAAATATTCTCCAATAGTACAACCTGAATAAGCTGATATAAATTGCATAGAAGCAGAATCAGAAGCTGTTGCAGCTACAATAATAGTATATGGCATTGCTCCATATTTTTCTAATAAATTTGCGGTTTTTTTAATTGATGATATCTTTTGTCCAATAGCAACATAAATACAAGTAACATTTTTATTTTTTTGATTAATAATTATGTCTATTGCTATGGAAGATTTTCCAGTTTGTCTATCTCCAATAATTAATTCACGTTGACCTTTTCCAATTGGCACTATAGAATCTATTGCTTTAATACCTGTTTGCAGGGGTTCGGAGACTGATTGTCGAGAAATTACTCCGGGAGCAACTTTTTCAATTGGAGCAGTTAATTTTGTTTTAATTGGCCCTTTTCCGTCAATTGGATCACCAAGAGCATTAATGACTCGACCACATAATTCCGGGCCAATAGGAACTTCTAAAATACGTCCTGTACATTTAACTATATCACCTTCCGTAATATGCT
